# Exhaled breath condensate pH as a biomarker of COPD severity in ex-smokers

**DOI:** 10.1186/1465-9921-12-67

**Published:** 2011-05-22

**Authors:** Andriana I Papaioannou, Stelios Loukides, Markos Minas, Konstantina Kontogianni, Petros Bakakos, Konstantinos I Gourgoulianis, Manos Alchanatis, Spyros Papiris, Konstantinos Kostikas

**Affiliations:** 1 Respiratory Medicine Department, University of Thessaly Medical School, Larissa, Greece; 2 2nd Respiratory Medicine Department, University of Athens Medical School, Athens, Greece; 3 1st Respiratory Medicine Department, University of Athens Medical School, Athens, Greece

## Abstract

Endogenous airway acidification, as assessed by exhaled breath condensate (EBC) pH, is present in patients with stable COPD. The aim of this study was to measure EBC pH levels in a large cohort of COPD patients and to evaluate associations with functional parameters according to their smoking status.

EBC was collected from 161 patients with stable COPD and 112 controls (current and ex-smokers). EBC pH was measured after Argon deaeration and all subjects underwent pulmonary function testing.

EBC pH was lower in COPD patients compared to controls [7.21 (7.02, 7.44) vs. 7.50 (7.40, 7.66); p < 0.001] and ex-smokers with COPD had lower EBC pH compared to current smokers [7.16 (6.89, 7.36) vs 7.24 (7.09, 7.54), p = 0.03]. In ex-smokers with COPD, EBC pH was lower in patients with GOLD stage III and IV compared to patients with stage I disease (p = 0.026 and 0.004 respectively). No differences were observed among current smokers with different disease severity. EBC pH levels in ex-smokers were associated with static hyperinflation (as expressed by IC/TLC ratio), air trapping (as expressed by RV/TLC ratio) and diffusing capacity for carbon monoxide, whereas no associations were observed in current smokers.

Endogenous airway acidification is related to disease severity and to parameters expressing hyperinflation and air trapping in ex-smokers with COPD. The possible role of EBC pH in COPD needs to be further evaluated in longitudinal studies.

## Introduction

Chronic obstructive pulmonary disease (COPD) is characterized by airflow limitation that is not fully reversible, is usually progressive, and is associated with an abnormal inflammatory response of the lungs in response to noxious particles and gases [1]. Although spirometry has been used as the gold-standard for the diagnosis and classification of COPD [1] as well as the marker for the description of disease severity, and progression [2], markers that express hyperinflation and low diffusing capacity seem to be also related to patients' mortality and health related quality of life [3]. Although many biomarkers have been proposed for the characterization of disease severity in COPD, none has been proven to be useful for that purpose yet [2].

It has been postulated that acidification of the exhaled air may be a surrogate marker of airway inflammation in COPD [4]. In support of this hypothesis we have previously shown an association of EBC pH with sputum neutrophils and markers of oxidative stress in exhaled air [5]. EBC pH has also been previously shown to be associated with FEV_1 _[5]. However, according to our knowledge, no study has evaluated the association of EBC pH with functional and clinical parameters that are relevant to clinical practice in a large cohort of patients with COPD.

The aim of the present study was to evaluate EBC pH levels in a large cohort of COPD patients with different disease severity and to compare them with smoking controls matched for age, gender and smoking habit. Associations of EBC pH values with functional parameters, including parameters expressing static hyperinflation and air-trapping, were evaluated. Finally, those associations were further analyzed according to the smoking status of COPD patients (current vs. ex-smokers).

## Materials and methods

### Study Subjects

From January 2008 to December 2009 we enrolled 161 consecutive COPD outpatients with mild to very severe airflow limitation (GOLD stage I to IV) and 112 control subjects (current and ex-smokers). All COPD study subjects had stable disease for the last 8 weeks. All subjects were evaluated in the outpatient COPD clinics of two tertiary University hospitals (University Hospital of Larissa and Attikon University Hospital of Athens). We excluded patients with other coexisting respiratory disorders, as well as patients with a history of smoking cessation <6 months, in order to stratify subjects as either current or ex-smokers. All patients provided a detailed history upon arrival, and underwent physical examination, exhaled breath condensate (EBC) collection and pulmonary function testing and, finally, bronchodilation testing and post-bronchodilation spirometry. Control subjects were otherwise healthy current or ex-smokers attending the outpatient clinics of the two hospitals for preoperative evaluation for elective minor surgery. None of our subjects had clinical history suggestive of gastroesophageal reflux disease. All subjects did not smoke or eat/drink any meal for at least 2 hours before collection. The study protocol was approved by the local ethics committees and all patients provided written informed consent.

### Pulmonary Function Tests

Pulmonary function tests (PFTs) were performed with a commercially available system (Master Screen, Erich Jaeger GmbH, Wuerzburg, Germany) and included post-bronchodilator forced expiratory volume in one second (FEV_1_), FVC, FEV_1_/FVC ratio, total lung capacity (TLC), residual volume (RV), inspiratory capacity (IC), and diffusing capacity for carbon monoxide (DL_CO_). Diffusing capacity for carbon monoxide (DL_CO_) and diffusing capacity for carbon monoxide adjusted for alveolar volume (DL_CO_/V_A_) were assessed by a single breath method with the patient in the sitting position. Lung function measurements were expressed as percentages of predicted values. Tests were performed according to the American Thoracic Society guidelines [6] by two experienced technicians, who were not aware of the EBC pH measurements, in order to ensure consistency of results.

### Exhaled Breath condensate collection and pH measurement

Exhaled breath condensate (EBC) was collected using a commercially available condenser (EcoScreen; Viasys, Hoechberg, Germany) according to the ATS/ERS recommendations [7]. Subjects rinsed their mouths with distilled water and were asked to perform tidal breathing for 20 min through a mouthpiece wearing a nose clip, while sitting comfortably on a chair. Approximately 2 mL of condensate were collected EBC pH was measured right after EBC collection as previously described [5,8]. Stable pH was achieved after deaeration with an inert gas (argon, 350 ml/minute for 10 minutes) and was measured using a commercially available pH meter (Model 3510, Jenway, Essex, UK).

### Statistical analysis

Data are expressed as mean ± standard deviation (SD) or as median (interquartile ranges) for normally distributed and skewed data, respectively. Normality of distributions was checked with Kolmogorov-Smirnov test. Comparisons between COPD patients were performed with Kruskal-Wallis tests for skewed variables and with one way analysis of variance (ANOVA) for normally distributed variables, with appropriate post-hoc tests (Dunn's and Bonferroni, respectively). Correlations were assessed using Spearman's rank correlation coefficient for skewed variables and Pearson's correlation coefficient for normally distributed variables. For the comparisons of EBC pH levels among groups with different pulmonary function tests severity, the study participants were divided to quartiles according to their results in the pulmonary function tests, with the exception of IC/TLC ratio where a previously reported cut-off point of ≤0.25 [9] was used for the expression of severe hyperinflation. P values < 0.05 were considered statistically significant. Statistical analysis was performed using the SPSS 16 statistical package (SPSS, Chicago, IL). Graphs were created using GraphPad Prism 5 (GraphPad Software Inc, La Jolla, CA, USA).

## Results

Demographic characteristics of the study subjects are shown in Table 1. Patients with COPD and controls did not differ in age, gender and smoking habit; however ex-smokers with COPD had significantly lower FEV_1_, FEV_1_/FVC and DL_CO _compared to current smokers.

**Table 1:** Demographic and functional characteristics of the study subjects

	Controls n = 112	COPD all n = 161	COPD ex-smokers n = 86	COPD current smokers n = 75
**Age**	65 ± 7	65 ± 10	68 ± 9	62 ± 10
**Gender M/F (n)**	88/24	122/39	62/24	60/15
**Pack-years**	61 ± 13	60 ± 14	64 ± 16	64 ± 22
**Current/ex-smokers (n)**	54/58	75/86		
**FEV**_**1 **_**(% pred.)**	93.8 ± 10.4	53.5 ± 17.7*	48.6 ± 17.3	59.2 ± 16.4^#^
**FEV**_**1**_**/FVC**	83.5 ± 9.8	56.8 ± 10.5*	54.0 ± 10.7	59.9 ± 9.4^#^
**IC/TLC**	0.72 ± 0.13	0.30 ± 0.09*	0.29 ± 0.09	0.30 ± 0.08
**RV/TLC**	0.35 ± 0.07	0.58 ± 0.11*	0.59 ± 0.12	0.56 ± 0.11
**DL**_**CO **_**(%pred.)**	87.8 ± 8.5	59.4 ± 22.2*	54.9 ± 23.8	64.1 ± 19.5^†^
**GOLD COPD stage (I/II/III/IV, n)**	N/A	16/81/44/20	5/37/28/16	11/44/16/4

Data are presented as mean ± standard deviation, unless otherwise indicated. FEV_1_: forced expiratory volume in 1 second, FVC: forced vital capacity, IC: Inspiratory capacity, TLC: total lung capacity, RV: residual volume, DL_CO_: diffusing capacity for carbon monoxide.

* p < 0.001 vs. controls, ^#^p < 0.001 vs. ex-smokers with COPD, ^†^p < 0.01 vs. ex-smokers with COPD

### EBC pH in Patients with Different Levels of COPD Severity

**Figure 1:**
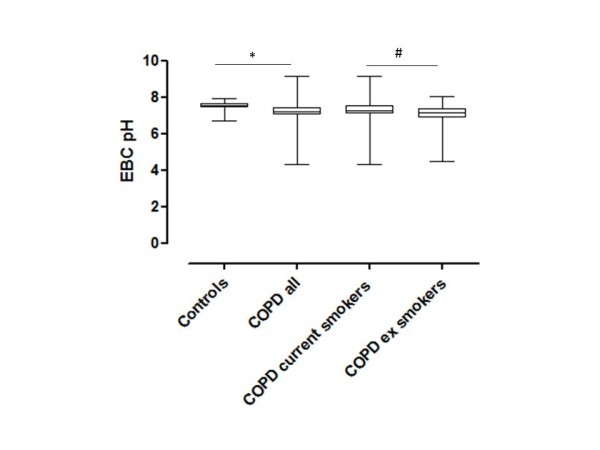
**EBC pH values in the 4 study groups of the whole population according to GOLD guidelines**. Box-plots represent median (interquartile ranges). * p < 0.001, # p < 0.01

**Figure 2:**
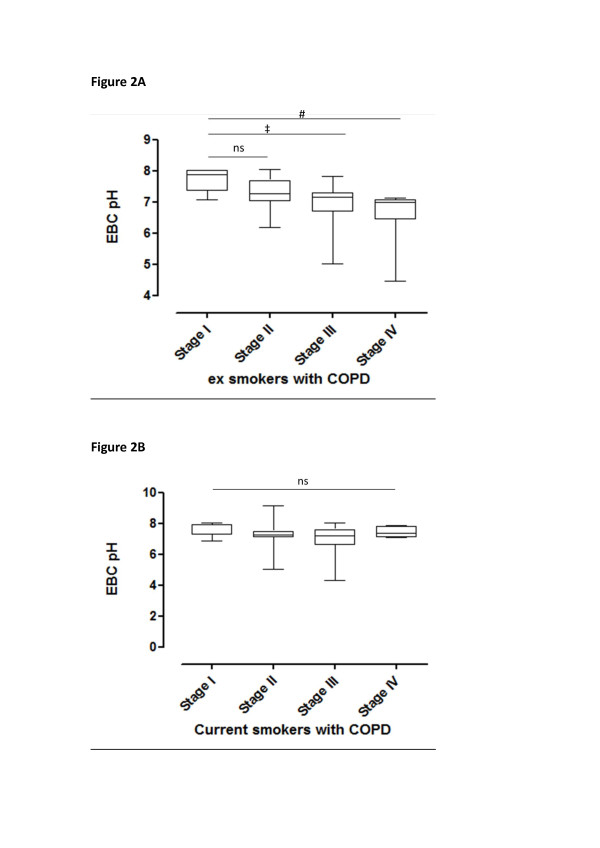
**EBC pH values according to COPD stages according to the GOLD guidelines**: **A. **in ex-smokers, **B. **in current smokers. Box-plots represent median (interquartile ranges). # p < 0.01, ‡ p < 0.05

EBC pH was significantly lower in patients with COPD compared to control subjects [7.21 (7.02, 7.44) vs. 7.50 (7.40, 7.66); p < 0.001]. Ex-smokers with COPD had lower EBC pH compared to current smokers with COPD [7.16 (6.89, 7.36) vs. 7.24 (7.09, 7.54), p = 0.03; Figure 1]. In ex-smokers with COPD, EBC pH was lower in patients with severe and very severe disease (GOLD stage III and IV) compared to patients with mild (GOLD stage I) disease 7.16 (6.70, 7.29) and 6.98 (6.45, 7.07) vs. 7.86 (7.36, 8.02), p = 0.026 and 0.004 respectively (Figure 2A)]. However, no differences were observed among COPD current smokers with different disease severity (Figure 2B).

### EBC pH Correlations

EBC pH presented significant correlations with several parameters of the pulmonary function tests of the COPD patients. Interestingly, those correlations were present only in the group of ex-smokers and were absent in the group of current smokers (Table 2).

**Table 2:** EBC pH correlations

	All		Ex-smokers		Current smokers	
	p-value	r	p-value	r	p-value	r
**FEV**_**1 **_**(%pred.)**	**<0.001**	**0.342**	**<0.001**	**0.452**	0.313	0.118
**FVC (%pred.)**	**0.001**	**0.265**	**<0.001**	**0.386**	0.944	0.008
**FEV**_**1**_**/FVC**	**<0.001**	**0.284**	**0.002**	**0.324**	0.064	0.215
**IC (%pred.)**	**0.05**	**0.200**	**0.025**	**0.299**	0.927	0.015
**TLC (%pred.)**	0.297	-0.105	0.392	-0.113	0.644	-0.075
**IC/TLC**	0.173	0.113	0.191	0.153	0.411	0.098
**RV (%pred.)**	**0.046**	**-0.200**	**0.044**	**-0.261**	0.462	-0.120
**RV/TLC**	**0.002**	**-0.303**	**0.006**	**-0.350**	0.212	-0.204
**DL**_**CO **_**(%pred.)**	**<0.001**	**0.304**	**<0.001**	**0.410**	0.536	0.074

FEV_1_: Forced expiratory volume in 1 second, FVC: forced vital capacity, IC: Inspiratory capacity, TLC: total lung capacity, RV: residual volume, DL_CO_: diffusing capacity for carbon monoxide

### Associations of EBC pH with Static Hyperinflation and Air-Trapping

EBC pH was significantly lower in COPD patients with severe hyperinflation, as expressed by an IC/TLC ratio ≤0.25 [7.19 (6.93, 7.32) vs. 7.23 (7.05, 7.53) respectively; p = 0.005. This difference was still present in ex-smokers [7.12 (6.89, 7.32) vs. 7.20 (7.00, 7.59); p = 0.024] whereas no statistical significant difference was observed in current smokers. EBC pH differed significantly between COPD patients with different severity of air trapping, as expressed by the RV/TLC quartiles (p < 0.001, Table 3). However, post hoc analysis revealed statistically significant difference only between the first and the fourth quartile (p < 0.001). This difference was observed in ex-smokers (p = 0.006), but not in current smokers (p = 0.398).

**Table 3:** EBC pH values according to RV/TLC quartiles in our study population

		**1**^**st **^**Quartile**	**2**^**nd **^**Quartile**	**3**^**rd **^**Quartile**	**4**^**th **^**Quartile**	p-value
**COPD all**	**RV/TLC**	≤0.50	0.50-0.58	0.58-0.66	>0.66	
	**EBC pH**	7.52 (6.88, 8.01)	7.19 (6.69, 7.69)	7.27 (7.08, 7.78)	6.93 (5.46, 7.19)^#^	<0.001
**COPD ex-smokers**	**RV/TLC**	≤0.52	0.52-0.60	0.60-0.68	>0.68	
	**EBC pH**	7.64 (6.88, 7.91)	7.10 (6.68, 7.45)	7.27 (7.08, 7.64)	6.92 (5.79, 7.07) ^†^	0.006
**COPD current smokers**	**RV/TLC**	≤0.47	0.47-0.55	0.55-0.63	>0.63	
	**EBC pH**	7.48 (7.06, 8.03)	7.36 (6.71, 7.95)	7.23 (6.47, 7.88)	7.13 (5.36, 7.84)	0.398

^#^p < 0.001 compared to the 1^st ^quartile

^†^p < 0.01 compared to the 1^st ^quartile

### Associations of EBC pH with Diffusing Capacity for Carbon Monoxide

EBC pH was significantly lower in patients with lower DL_CO _(p = 0.0006, Table 4). This difference was present in ex-smokers (p = 0.004) but not in current smokers (p = 0.82).

**Table 4:** EBC pH values according to DL_CO _(%pred.) quartiles in our study population

		**1**^**st **^**Quartile**	**2**^**nd **^**Quartile**	**3**^**rd **^**Quartile**	**4**^**th **^**Quartile**	p-value
**COPD all**	**DL**_**CO**_	≤45.2	45.2-60.0	60.0-72.0	>72.0	
	**EBC pH**	7.08 (6.76, 7.50)	7.09 (6.69, 7.24)	7.30(7.13, 7.69) ^‡^	7.28 (7.13, 7.28)	0.006
**COPD ex-smokers**	**DL**_**CO**_	≤35.8	35.8-54	54-70.2	>70.2	
	**EBC pH**	7.04 (6.07, 7.38)	7.08 (6.79, 7.24)	7.19 (7.03, 7.32)	7.37 (7.19, 7.86)^†^	0.004
**COPD current smokers**	**DL**_**CO**_	≤53.8	53.8-67.3	67.3-72.8	>72.8	
	**EBC pH**	7.21 (6.71, 7.76)	7.25 (7.09, 7.86)	7.23 (7.11, 7.69)	7.25 (7.10, 7.34)	0.82

^‡^p < 0.05 compared to the 1^st ^quartile

^†^p < 0.01 compared to the 1^st ^quartile

## Discussion

In the present study we have shown that EBC pH levels are lower in patients with COPD compared to age and gender matched controls and are related to disease severity, as expressed by GOLD stages, especially in ex-smokers. EBC pH levels are additionally associated with the presence of static hyperinflation (as expressed by the IC/TLC ratio), air trapping (as expressed by the RV/TLC ratio) and diffusing capacity for carbon monoxide, and those associations were evident in ex-smokers, whereas no similar associations were observed in current smokers with COPD. This is the first study to our knowledge that evaluated the associations of EBC pH in a large cohort of COPD patients, suggesting that it may represent a biomarker of disease severity in ex-smokers with COPD.

EBC pH represents a surrogate marker of airway acidification that has been associated with the presence of acidification in all levels of the respiratory tract [10]. Airway acidification has been reported to cause bronchoconstriction [11], impaired ciliary motility [12], increased mucous production and viscosity [13], and airway epithelial damage [14], that may contribute to the development of airway disease.

We have observed that COPD patients with more severe disease and more severe functional impairment have lower EBC pH, which may reflect more intense airways inflammation related to the presence of acidification. Additionally, ex-smokers had significantly lower EBC pH values compared to current smokers with COPD. A possible explanation for the lower EBC pH values in ex-smokers may be the fact that these patients had more severe disease compared to current smokers, as expressed by the significantly lower FEV_1_, FEV_1_/FVC and DL_CO _compared to current smokers. Moreover, there is evidence that airway inflammation is present in COPD patients even after smoking cessation [15]. Interestingly, in a longitudinal study involving bronchial biopsies in patients with COPD, the number of sputum neutrophils, lymphocytes, interleukin-8 and eosinophilic-cationic-protein levels significantly increased at 12 months after successful smoking cessation [15], whereas in a cross-sectional study with bronchial biopsies, ex-smokers with COPD had higher CD3+, CD4+, and plasma cell numbers compared to current smokers [16]. The above data may further explain the significant differences in EBC pH levels between current and ex-smokers with COPD.

The presence of significant associations of EBC pH with functional parameters related to disease severity in ex-smokers with COPD in our study, in contrast to current smokers, is supported by previous findings suggesting that the inflammatory effects of current smoking may mask the underlying ongoing inflammatory process of COPD [17]. Smoking causes an acute oxidative burst in the airways of current smokers [18], and therefore a plausible explanation may be the fact that in current smokers such associations may have been obscured by current smoking. A previous study by Borrill et al. failed to show any associations between EBC pH levels and FEV_1 _[19]. That study included a significantly smaller number of COPD patients (n = 36) compared to the present one, thus it may have not been adequately powered to detect such an association. Despite that, those authors were able to find a statistically non-significant trend in ex-smokers and suggested that a larger study is needed. In the present study, in a larger population, we have shown a significant association between FEV_1 _and EBC pH that was present only in ex-smokers, after the elimination of the confounding role of continuing smoking.

In this study, we have used as a cut-off point for the expression of severe hyperinflation, a value of the IC/TLC ratio ≤0.25, as this cut-off point is considered to express severe hyperinflation and has been associated with increased mortality in COPD [9]. A recent study has reported a logarithmic correlation of EBC nitrite and pulmonary function tests indicating hyperinflation for COPD patients classified in GOLD class 2 or more. In contrast, EBC nitrite level was not correlated with airway obstruction or proinflammatory cytokines [20]. On the basis of this report, it has been recently suggested that the estimation of the extent of hyperinflation in studies involving COPD patients may lead to the more proper interpretation of EBC biomarker levels compared to airflow limitation [21]. In the present study, we have observed that ex-smokers with COPD and severe static hyperinflation have lower EBC pH levels, further supporting a role of EBC pH as a biomarker in this subgroup of patients.

In a similar manner, we have shown that COPD patients with severe air-trapping and severe impairment of DL_CO_, as characterized by the lower quartiles of those parameters, presented significantly lower EBC pH values and this was more evident in ex-smokers. It has been previously reported that in the small airways of patients with severe COPD there is an increased number of leucocytes which is correlated with reduced expiratory flow, lung hyperinflation and carbon monoxide diffusion impairment, suggesting that the inflammatory response plays a significant role in the clinical progression of the disease [22].

The independent association of decreased pH values with parameters expressing hyperinflation is a novel finding of the present study, suggesting that this may represent an inflammatory component related to lung mechanics. In a well characterized population of COPD patients, researchers from the GLUCOLD study group have shown that patients with emphysema present differences in sputum cell counts (i.e. higher neutrophil and lower eosinophil counts), compared to patients with chronic bronchitis [23]. The patients with hyperinflation and lower DL_CO _in our population may well represent patients with predominance of emphysema. Moreover, our group has previously shown that EBC pH is inversely related with sputum neutrophil counts [5]. Taken together, these data may suggest a possible link between hyperinflation, emphysema and airways acidification.

Although several factors can affect EBC pH, including the effect of drinking or eating [24], smoking [25,26], the presence of extrapulmonary components [27], the dilution bias and the purging procedure in order to achieve the stability of the sample, we tried to overcome all the above issues by avoiding eating and drinking for at least 2 hours before collection, by excluding patients with overt gastroesophageal reflux disease and, finally, by following the current standardized procedure for pH collection and measurement [7]. Moreover, the careful characterization and the large number of COPD patients included in the present study further support the validity of our results. Another possible limitation is the large variation in the measurements of EBC pH that leads to a large overlap between study groups, and this may limit the clinical utility of measuring EBC pH in individual patients. However, we believe that the division of the patients into subgroups according to the presence of hyperinflation, air trapping and/or reduced diffusing capacity in the present study has significantly reduced variation and provides possible implications for clinical application of this biomarker.

In conclusion, in the present study we have shown that EBC pH is lower in COPD patients compared to otherwise normal smokers. Moreover, we have shown that EBC pH is related to disease severity, and to parameters expressing airflow limitation, hyperinflation and air trapping. Those correlations were more robust in ex-smokers while no significant correlations were found in current smokers. Our findings suggest that endogenous airway acidification, as expressed by EBC pH, may be an indicator of disease severity in ex-smokers with COPD. In contrast, the above observations may lead to the hypothesis that current smoking in COPD patients may influence and alter the results of chronic inflammation, therefore rendering the interpretation of exhaled biomarkers more difficult. Longitudinal studies evaluating the effect of therapeutic interventions targeting lung hyperinflation and/or air-trapping on EBC pH in ex-smokers with COPD are justified by our results.

## Competing interests

The authors declare that they have no competing interests.

## Authors' contributions

KK and SL conceived of the study, and participated in its design and coordination. AIP, MM, KKon and PB performed the recruitment of patients and study measurements. KK, AIP and MM performed the statistical analysis. AIP and KK drafted the manuscript. KIG, MA, SP and SL were involved in revising the manuscript for important intellectual content. All authors read and approved the final manuscript.
